# The impact of genetic merit on ewe performance and efficiency parameters

**DOI:** 10.1093/jas/skab301

**Published:** 2021-10-21

**Authors:** Nicola Fetherstone, Noirin McHugh, Tommy M Boland, Philip Creighton, Norann Galvin, Fiona M McGovern

**Affiliations:** 1 Animal & Grassland Research and Innovation Centre, Teagasc, Mellows Campus, Athenry, Co. Galway, H65 R718, Ireland; 2 School of Agricultural Science, University College Dublin, Belfield, Dublin 4, D04 V1W8, Ireland; 3 Animal & Grassland Research and Innovation Centre, Teagasc, Moorepark, Fermoy, Co. Cork, P61 C996, Ireland

**Keywords:** body condition score, body weight, efficiency, feed intake, milk

## Abstract

The aim of this study was to investigate the impact of ewe genetic merit on ewe performance and efficiency parameters. The study consisted of three genetic merit groups (New Zealand [**NZ**], High Irish, and Low Irish) and ran from 2016 to 2019, inclusive. Each genetic merit group contained 30 purebred Suffolk and 30 purebred Texel ewes, which were selected based on their maternal genetic indexes in their country of origin, namely Ireland (€uro-star Replacement index) or New Zealand (New Zealand Maternal worth). Ewe body condition score (**BCS**), ewe body weight (**BW**), milk yield, milk composition, dry matter intake (**DMI**), and efficiency parameters were all analyzed using linear mixed models. Ewe BW was similar across all genetic merit groups at each time point (*P* > 0.05). In comparison to both High and Low Irish ewes, NZ ewes had a higher BCS at mating, mid-pregnancy, lambing, week 10 post-lambing (**PL**, *P* < 0.05). Ewe BW change was similar across genetic merit groups, except between mating and mid-pregnancy where ewe BW loss was greater for NZ ewes than Irish ewes (*P* < 0.05) and between weeks 6 PL and 10 PL, where NZ ewes gained BW and High and Low Irish ewes lost BW (*P* < 0.01). Ewe milk yield, milk fat, total solids, and gross energy content were superior for milk produced by NZ ewes at week 6 PL in comparison to milk produced by High Irish and Low Irish ewes (*P* < 0.01). NZ ewes produced a greater quantity of milk solids/kg of BW at week 6 PL compared with High Irish ewes (*P* < 0.01), whereas Low Irish ewes did not differ from either NZ or High Irish (*P* > 0.05). Low Irish ewes had a greater daily DMI than High Irish ewes in late lactation (week 10 PL, *P* < 0.05) and had a greater DMI/kg of ewe BW compared with the High Irish ewes at the same time point (*P* < 0.05). NZ ewes weaned a litter BW equivalent to 60.4% of their mating BW, which was more than the Low Irish ewes who weaned 57.1% of the ewe’s BW at mating (*P* < 0.01), whereas the High Irish ewes did not differ from either the NZ or Low Irish ewes at 59.3% of the ewe’s BW at mating (*P* > 0.05). This study presents a range of parameters across ewes of high and low genetic merit, demonstrating the ability to achieve gains through selection of animals of high genetic merit. Sheep producers should consider genetic indexes as a tool to assist in the decision-making process of selecting replacement ewes and/or breeding rams, once satisfied the animal is correct, and meeting the breeding objectives of the system.

## Introduction

The global population is expected to reach more than 9 billion people by 2050, thus placing increased pressure on the agricultural industry to improve the productivity, efficiency, and sustainability of its systems (Boland et al., 2013). For sheep, more efficient production through increased lamb output per ewe has been discussed widely (Duguma et al., 2002; Keady et al., 2009; McHugh et al., 2018). Some studies, including Cloete and Durand (1994), McEwan et al. (2001), and Lambe et al. (2014), have identified potential in maternal productivity and efficiency traits including number of lambs weaned per ewe joined. Other previous studies have shown significant potential to increase lamb carcass output per ewe and per hectare, through increased stocking rates or prolificacy potential (Ho et al., 2014; Kilcline et al., 2015; Earle et al., 2017a). Investigation into other efficiency parameters such as kilogram dry matter intake (**DMI**), milk yield produced, or milk solids produced/kg ewe body weight (**BW**) within the sheep industry is limited, particularly when recorded simultaneously, in contrast to beef and dairy systems (Beukes et al., 2010; McCabe et al., 2017; O′Sullivan et al., 2020).

The superiority of high genetic merit ewes, in comparison to low genetic merit ewes, has previously been demonstrated across many reproductive, lambing, and lamb performance traits (Lewis et al., 1996; Márquez et al., 2013; Fetherstone et al., 2021c), and the potential benefits to industry from their strategic use have been discussed (Fetherstone et al., 2021a). However, to the authors’ knowledge, few studies, if any, have simultaneously recorded this variety of ewe performance and novel efficiency traits within a production system study in the past. Furthermore, the comparison of Irish and New Zealand sheep genetics within the same environment and the comparison of sheep divergent on genetic merit for maternal traits in the national Irish breeding objectives as part of a production system study have not been reported to date. The objective of this study, therefore, was to quantify the impact of ewe genetic merit on a plethora of ewe efficiency and performance parameters.

## Materials and Methods

### Study design

This study was performed at Teagasc, Animal and Grassland Research Center, Mellows Campus, Athenry, Co. Galway, Ireland (54° 80′ N; 7°25′ W) over a 4-yr period from 2016 to 2019. All procedures were conducted under approval from the Teagasc Animal Ethics Committee on experimental animal use (TAEC56-2014) and the Health Protection Regulation Authority (AE19132/P039) in accordance with the Cruelty to Animals Act 1876 and the European Communities Regulations, 1994.

Three genetic groups of ewes were selected in October 2015: high maternal genetic merit ewes of New Zealand origin (**NZ**), high maternal genetic merit ewes of Irish origin (High Irish), and low maternal genetic merit ewes of Irish origin (Low Irish; Fetherstone et al., 2021c). Each of the three groups consisted of 30 purebred Suffolk ewes and 30 purebred Texel ewes. A cohort of NZ Suffolk and Texel ewes had previously been imported into Ireland in 2013 and 2014 and were selected from within the top 40% across breed for maternal traits (Byrne et al., 2012) based on the New Zealand Maternal Worth Index, with an average genetic merit value of –NZ$1.48. Irish ewes were selected based on their Irish €uro-star Replacement index (Bohan et al., 2019) and were classified as either High Irish (top 20% within breed) or Low Irish (bottom 20% within breed). The importation of high maternal genetic merit NZ ewes was performed in an attempt to quantify their performance relative to their Irish contemporaries; the selection of Irish ewes divergent on genetic merit for maternal traits was to ensure that the national breeding objectives are improving the performance and productivity of the national flock. The average Irish €uro-star Replacement index for each of the three genetic groups at the start of the study was €0.06 ± 0.74, €1.04 ± 0.62, and −€0.68 ± 0.73 for the NZ, High Irish, and Low Irish genetic groups, respectively. In total, data were recorded on 350 individual ewes and their lambs over the period of the study. The study consisted of primiparous (proportion in parenthesis; 0.25) and multiparous ewes in first (0.30), second (0.22), third (0.15), fourth (0.07), and fifth (0.01) parity ewes.

Ewes were synchronized and mated to rams within the same genetic merit group and breed via laparoscopic artificial insemination (**AI**) during the first and second weeks of October each year. Ewes that failed to conceive to AI were naturally mated to rams from the same genetic merit group and breed within 21 d of AI. Housing took place in early December with ewes offered grass silage ad libitum. Ewes were pregnancy scanned in early January. Ewes recorded as barren at scanning were removed from the genetic merit group at that time point. Post-scanning, ewes were penned in groups according to genetic merit group and litter size, and concentrate feeding was then calculated on the number of ewes per pen basis, silage quality, and ewe energy requirements according to litter size (Alderman and Cottrill, 1996) from week 8 prior to the predicted lambing date. In total, 4.2, 24.5, and 29.4 kg of concentrate were offered to single, twin, and triplet/quad ewes, respectively; concentrate feed levels started at 150 g/d at 8 wk pre-lambing and increased incrementally until the maximum feeding rate of 1 kg/d was reached for triplet-bearing ewes at lambing.

Lambing commenced in the last week of February, with a mean lambing date of March 8. Lamb BW (kg) was recorded at birth, week 6 post-lambing (**PL**), and fortnightly thereafter. The maximum number of lambs reared by a ewe was two; in ewes with a litter size of three or greater, surplus lamb(s) were either artificially reared or cross-fostered to a dam within the same genetic merit group. Artificially reared lambs were omitted from all further analyses and cross-fostered lambs were assigned to their foster dam. Post-lambing, each genetic merit group of ewes and their lambs was turned out onto a perennial ryegrass (*Lolium perenne*) and white clover (*Trifolium repens*) sward at a stocking rate of 12 ewes/ha in separate farm-lets. Each genetic merit group was allocated 5 ha, distributed equally across the farm-let, that is, four paddocks (rotation 1) or eight subdivided paddocks (rotation 2 onwards). A rotational grazing system was operated whereby each genetic merit group (ewes and lambs) grazed the paddocks assigned to them at the start of the study, for the duration of the study. Pre-grazing heights range from 7 to 9 cm. Post-grazing heights were 3.5 cm for the first rotation, and 4.1 cm thereafter, as in line with previously reported guidelines (Earle et al., 2018). Concentrate feeding ceased on the return of ewes to grazing PL, with the exception of 2018 when adverse weather conditions resulted in supplementary feeding being required for 6 wk. Any ewe that failed to rear a lamb was removed from the genetic merit group and replaced by another of similar status in order to maintain a similar stocking rate per hectare across each genetic merit group.

### Animal measurements

#### BW and BCS

Ewe BW (kg) was measured using Prattley weigh scales (Prattley Industries Ltd., Temuka, New Zealand) and Tru-test XR3000 (Tru-test, Auckland, New Zealand), at increments of 0.5 kg. Body condition score (**BCS**) was measured on a scale of 1 to 5, in increments of 0.25 (adapted from Russel et al., 1969), and was measured by the same technician for the duration of the experiment. Both ewe BW and BCS (*n* = 180 ewes per year) were measured at six time points throughout each production year: pre-mating, mid-pregnancy (coinciding with pregnancy scanning), lambing (i.e., at a maximum of 24 h PL), and at weeks 6, 10, and 14 PL (i.e., weaning). The change of BW and BCS between consecutive time points of each ewe was also calculated. Litter BW at weaning expressed as a percentage of ewe BW at mating was used as an estimate of ewe efficiency.

#### Milk yield and composition

The average daily milk yield on a subset of ewes (*n* = 10 ewes per genetic merit group, i.e., a total of 30 ewes per year were selected and balanced for breed, rearing litter size, and parity) was estimated using the weigh-suckle-weigh technique (Doney et al., 1979), twice weekly at weeks 4 and 6 PL annually. The weigh-suckle-weigh procedure involved a 3-h separation period of the ewes and lambs after which lambs were weighed before and after suckling, and the weight difference as well as any surplus milk was collected (hand-milked) and used to calculate individual ewe milk yield. Milk composition was estimated from a 15-mL sample from each of the 30 ewes per milking event each year. Samples were analyzed using a Milkoscan FT 6000 (Foss Electric DK-3400, Hillerod, Denmark) for milk fat, protein, lactose, and total milk solids (**MS**). Gross energy content of the milk sample taken from each ewe (KJ/kg) was then calculated as described by Šebek and Everts (1993). Milk efficiency parameters reported included the total volume of milk and MS produced per kilogram of ewe BW at week 6 PL.

#### Dry matter intake

Daily DMI (kg DM/[ewe ⋅ day]) was measured on 18 ewes per genetic merit group (balanced for breed, litter size, and parity, while also aiming to avoid the selection of ewes that already took part in the milk yield and composition experiment) at three time points: early (week 5 PL) and late (week 10 PL) lactation, and during the dry period (week 23 PL) annually. DMI was also reported relative to the BW of the ewe at the time of measurement as an indication of ewe efficiency. DMI was estimated on a per ewe basis using the *n*-alkane technique as described by Dove and Mayes (1996) and validated for grazing sheep by McGovern et al. (2020). In summary, an *n*-alkane bolus containing 132 mg of C32-alkane (*n*-dotriacontane) was administered to each ewe for 11 consecutive days, while fecal samples were collected and analyzed from day 6 to 12. Herbage samples, that represented the herbage available for grazing, were harvested from day 6 to 11. Samples were bulked to give one sample/(ewe ⋅ intake period). The ratio of herbage C33 alkane (tritriacontane) to dosed C32 alkane (*n*- dotriacontane) was used to predict ewe DMI.

### Statistical analysis

The effect of genetic merit (NZ, High Irish, or Low Irish) on ewe BW and BCS and their change over time (*n* = 725), milk yield (*n* = 477), milk composition (*n* = 230), gross energy content (*n* = 230), and DMI (*n* = 538) were analyzed using linear mixed models in PROC Mixed (SAS Inst. Inc., Cary, NC), with ewe genetic merit (NZ, High Irish, or Low Irish), ewe breed (Suffolk or Texel), ewe parity (1, 2, 3, 4, or ≥5), and date of trait measurement included as fixed effects for all models. When the traits under investigation fell within the preweaning period, days since lambing, litter birth (1, 2, 3, or 4), and rearing type (1 or 2) were also included as fixed effects in the models. Ewe within year was included as a repeated effect for milk and DMI parameters. Sire of the dam within strain (*n* = 127) was included as a random effect for all models, whereas sire of the lamb was included for lamb measures (*n* = 107 sires). All parameters were recorded either on a per ewe basis or on a per litter basis and reported as an average of the experimental group.

## Results

### Ewe BW and BCS

**Figure 1.**
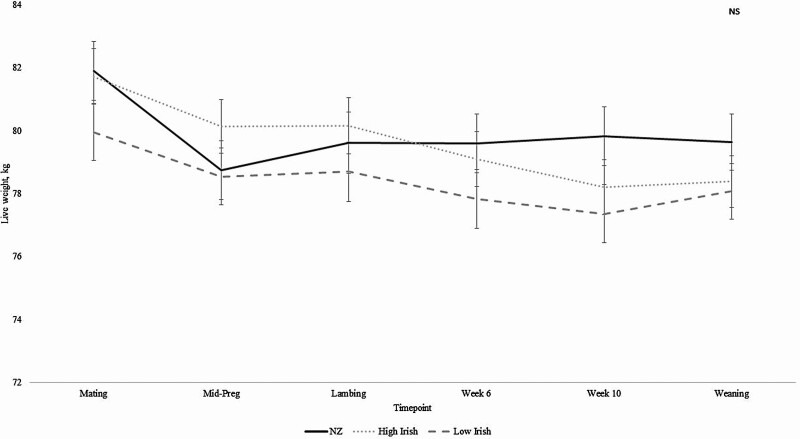
The effect of genetic merit (New Zealand [NZ], High Irish, and Low Irish) on ewe body weight (BW, kg; SEM included in error bars) at given time points throughout the year (NS = *P* > 0.05).

Ewe BW (kg) was similar across genetic merit groups at every time point (Figure 1; *P* > 0.05). NZ, High Irish, and Low Irish ewes were heaviest at mating, with weights of 81.90, 81.72, and 79.96 kg, respectively (Figure 1; *P* > 0.05). NZ ewes lost the most BW from mating to mid-pregnancy compared with either Irish group (Table 1; *P* < 0.05). NZ ewes were at their lightest at mid-pregnancy (78.75 kg), while BW change between mid-pregnancy and lambing and between lambing and week 6 PL was similar across all three groups (Table 1; *P* > 0.05). High and Low Irish ewes reached their lightest (78.21 and 77.36 kg, respectively) at week 10 PL (Figure 1). Ewe BW change between week 10 PL and weaning was similar across each of the genetic merit groups (Table 1; *P* > 0.05). Furthermore, although the greatest change in BW occurred between weaning and the following mating (range 6.32 to 8.02 kg), there was no significant difference between the three genetic merit groups (Table 1; *P* > 0.05).

**Table 1.** The effect of genetic merit (New Zealand [NZ], High Irish, and Low Irish) on the change of ewe body weight (BW, kg) and body condition score (BCS) over time

	Genetic merit group^2^			SEM	*P*-value
	NZ	High Irish	Low Irish		
BW change, kg					
Mating to mid-pregnancy	−2.59^a^	−1.15^b^	−1.48^b^	0.403	<0.05
Mid-pregnancy to lambing	1.10	−0.06	0.09	0.504	NS
Lambing to week 6 PL^1^	−0.16	−1.13	−0.22	0.534	NS
Week 6 to 10 PL^1^	0.33^a^	−0.74^b^	−0.96^b^	0.310	<0.01
Week 10 PL^1^ to weaning	−0.24	0.16	0.32	0.517	NS
Weaning to next mating	8.02	7.76	6.32	0.662	NS
BCS change					
Mating to mid-pregnancy	−0.08^a^	−0.01^ab^	0.01^b^	0.030	0.05
Mid-pregnancy to lambing	−0.39	−0.41	−0.44	0.033	NS
Lambing to week 6 PL^1^	−0.09^a^	−0.03^a^	0.07^b^	0.032	<0.01
Week 6 to 10 PL^1^	0.09	0.09	0.04	0.023	NS
Week 10 PL^1^ to weaning	−0.06^a^	0.03^b^	0.00^ab^	0.029	<0.05
Weaning to next mating	0.49^a^	0.28^b^	0.28^b^	0.036	<0.001

^1^PL, weeks post-lambing.

^2^Results are least-squares means resulting from the multiple mixed linear regressions.

^a,b^Within a row, means with a common superscript do not differ (*P* > 0.05).

**Figure 2.**
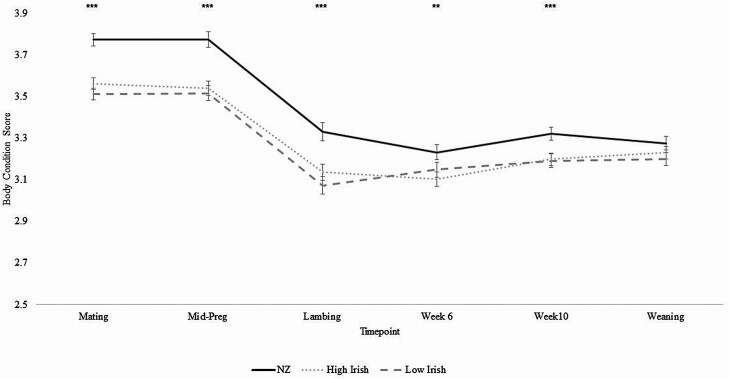
The effect of genetic merit (New Zealand [NZ], High Irish, and Low Irish) on ewe body condition score (SEM included in error bars) at given time points throughout the year. Significant differences between genetic merit groups are displayed as ***P < 0.001 and **P < 0.01 at a given timepoint.

BCS for NZ ewes was greater than both High and Low Irish ewes at mating, mid-pregnancy, lambing, and week 10 PL (Figure 2; *P* < 0.01). No differences in BCS of High and Low Irish ewes were observed throughout the year (Figure 2; *P* > 0.05). The greatest BCS was achieved at mating (3.56) and mid-pregnancy (3.54) for NZ, High Irish, and Low Irish ewes, respectively (Figure 2). At week 6 PL, a greater BCS was observed in NZ ewes (3.23) relative to High Irish ewes (3.10, Figure 2; *P* < 0.01); Low Irish ewes did not differ from either the NZ or High Irish (Figure 2; *P* > 0.05). At weaning, no differences in BCS were observed between any of the genetic merit groups (Figure 2; *P* > 0.05).

The greatest decline in BCS for all of the genetic merit groups occurred between mid-pregnancy and lambing but did not differ between any of the genetic merit groups (Table 1; *P* > 0.05). Low Irish ewes began to gain condition after lambing, gaining +0.07 of a condition score between lambing and week 6 PL, unlike the NZ and High Irish groups that utilized body reserves and lost condition during the same period, albeit differences were biologically small (Table 1; *P* < 0.01). The greatest difference between the genetic merit groups was observed for the change of BCS between weaning and the next mating, where NZ ewes gained +0.21 of a condition score more than both the High and Low Irish ewes (Table 1; *P* < 0.001).

### Proportion of litter BW weaned per ewe relative to her own BW at mating

NZ ewes weaned a litter BW equivalent to 60.4% of the ewe’s BW at mating—a greater percentage of maternal weight weaned in comparison to Low Irish ewes (57.1 %, *P* < 0.01)—while the High Irish ewes did not differ from either the NZ or the Low Irish ewes weaning litter BW equivalent to 59.3% of their BW at mating.

### Milk yield and composition

Milk yield and milk composition results are presented in Table 2. At week 4 PL, ewes of high genetic merit, regardless of country of origin, had a greater milk yield in comparison to the Low Irish ewes (*P* < 0.05). At week 6 PL, although the average daily milk yield declined, greater milk yields were associated with the NZ ewes relative to both the High and Low Irish ewes (*P* < 0.01). The fat percentage of milk from NZ, High Irish, and Low Irish ewes was similar at week 4 PL (*P* > 0.05) but higher for the NZ ewes at week 6 PL, compared with either the High or Low Irish ewes (*P* < 0.001). Milk from High Irish ewes had a lower protein percentage in comparison to NZ and Low Irish ewes at week 4 PL (*P* < 0.01), while no difference was reported between the ewes at week 6 PL (*P* > 0.05). Lactose content of the milk was similar for NZ, High, and Low Irish ewes at both time points (*P* > 0.05). Total milk solids and gross energy content of the milk were greater for NZ ewes in comparison to either High or Low Irish ewes at week 6 PL (*P* < 0.01). The quantity of milk expressed per unit of ewe BW at week 6 PL was similar for NZ, High, and Low Irish ewes (*P* > 0.05) and ranged from 0.032 (High Irish and Low Irish) to 0.038 (NZ) kg milk yield/kg ewe BW. However, when production was expressed as a quantity of MS produced per kilogram BW at week 6 PL, rather than the quantity of milk produced, NZ ewes produced (0.229 kg MS/kg BW) more than High Irish ewes (0.198 kg MS/kg BW; *P* < 0.01), whereas Low Irish ewes were intermediate (0.215 kg MS/kg BW) and did not differ from either the NZ or High Irish ewes (*P* > 0.05).

**Table 2.** The effect of genetic merit (New Zealand [NZ], High Irish, and Low Irish) on ewe milk yield, milk composition, and energy content

	Genetic merit group^2^			SEM	*P*-value
	NZ	High Irish	Low Irish		
Week 4 PL^1^					
Milk yield, kg	3.56^a^	3.48^a^	2.99^b^	0.298	<0.05
Milk fat, %	7.20	7.62	6.70	0.543	NS
Protein, %	5.23^a^	4.88^b^	5.38^a^	0.133	<0.01
Lactose, %	5.11	5.08	5.08	0.072	NS
Total solids, %	18.05	18.17	17.72	0.508	NS
Week 6 PL^1^					
Milk yield, kg	2.98^a^	2.50^b^	2.38^b^	0.163	<0.01
Milk fat, %	7.15^a^	5.73^b^	5.23^b^	0.351	<0.001
Protein, %	5.65	5.13	5.44	0.191	NS
Lactose, %	4.98	4.77	5.04	0.110	NS
Total solids, %	18.56^a^	16.45^b^	16.44^b^	0.457	<0.01
Gross energy content of milk					
Week 4, KJ/kg	4,942	5,062	4,753	223.06	NS
Week 6, KJ/kg	4,962^a^	4,236^b^	4,131^b^	159.83	<0.01

^1^PL,weeks post-lambing.

**2**Results are least-squares means resulting from the multiple mixed linear regressions.

^a,b^Within a row, means with a common superscript do not differ (*P* > 0.05).

### Dry matter intake

**Figure 3.**
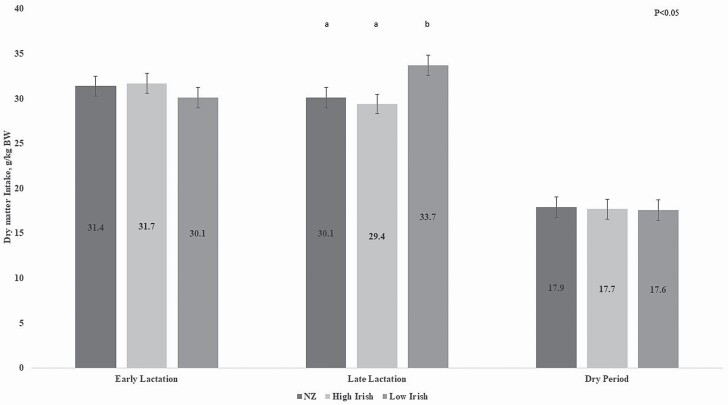
The effect of genetic merit (New Zealand [NZ], High Irish, and Low Irish) on ewe dry matter intake/kg of ewe body weight (BW; SEM included in error bars). ^a,b^Least square means with different superscripts differ (*P* < 0.05) from each other.

The average estimated daily DMI in early lactation (week 5 PL) did not differ by genetic merit group and was 2.44, 2.41, and 2.31 kg DM/d for NZ, High Irish, and Low Irish ewes, respectively (SEM ± 0.087; *P* > 0.05). By late lactation (week 10 PL), Low Irish had a greater intake than the High Irish ewes (*P* < 0.01). There was no difference in the daily DMI of NZ, High Irish, or Low Irish ewes during the dry period (week 23 PL) with intakes of 1.54, 1.48, and 1.50 kg DM/d (*P* > 0.05). When DMI was displayed as a proportion of ewe BW (g DMI/kg BW), a similar ratio was observed between all genetic merit groups in early lactation and in the dry period (Figure 3; *P* > 0.05). However, in late lactation, Low Irish ewes consumed 13% more DMI per kilogram BW than the High Irish ewes (*P* < 0.05) and consumed 11% more DMI per kilogram BW than the NZ ewes (Figure 3; *P* < 0.1).

## Discussion

As the demand for efficient agricultural production systems increases, the agricultural industry must identify traits for selection that can improve efficiency in order to achieve future global food security targets (Berry and Crowley, 2013). Using animals of high genetic merit, regardless of species, has previously been reported as a method of accelerating farm production gains (Ramsbottom et al., 2011; Márquez et al., 2013; McHugh et al., 2014). Previous studies showed that the feed and production efficiency of beef cattle (Kelly et al., 2020) and the reproductive efficiency and survival of dairy cows (O′Sullivan et al., 2020) could be enhanced through the selection of animals based on their genetic merit. Other studies have reported ewe efficiency measures on specific parameters such as BCS (Corner-Thomas et al., 2015), BW (Kenyon et al., 2004, 2009), milk yield (Cardellino and Benson, 2002), estimates for DMI at pasture (Earle et al., 2017a), and lamb output (McHugh et al., 2018), but to the authors’ knowledge to date, an array of these types of production efficiency measures for ewes has not been evaluated simultaneously to date. Although not an objective of the study, each genetic merit group was balanced by breed (Suffolk and Texel), in order to avoid the confounding of genetic effects. While results across breed were not reported within this paper, they were calculated as part of the statistical analysis (least square means), which indicate similarities within breed across a range of traits including BW at mating (83.18 and 83.22 kg for High Suffolk and Texel, respectively) and BCS at weaning (3.16 and 3.18 for Low Suffolk and Texel, respectively). Results from this study highlight areas where differences in performance can be achieved through selection of genetic merit of the ewe, for example, milk yield in early lactation, DMI in late lactation, and BCS change in early lactation.

BCS is known to influence both reproductive and production performance across both sheep (Keady et al., 2009; Kenyon et al., 2014), beef cows (Bohnert et al., 2013), and dairy cows (Pryce et al., 2001). In dairy, cows of NZ origin have been shown to maintain a higher BCS throughout the production year, in comparison to those of Irish origin (Horan et al., 2005; McCarthy et al., 2007). In the current study, changes in BCS across all genetic merit groups throughout the production year were biologically small but were similar to results previously reported within similar production system studies (Earle et al., 2017b; Macé et al., 2018). Surprisingly, the greatest change in BCS between two time points occurred between mid-pregnancy and lambing, indicating a need to review late pregnancy nutritional management, but this change was somewhat similar to the −0.3 change observed within a similar production system study by Higgins et al. (2020). Pryce et al. (2001) highlighted that as genetic merit for milk production increases, so too does the mobilization of body reserves; similarly, within the current study, ewes of high genetic merit mobilized reserves between lambing and week 6 PL, whereas Low Irish animals gained condition, albeit at low levels. This may be a contributing factor to the results reported previously by Fetherstone et al. (2021c) who highlighted the ability of the ewes of high genetic merit, whether NZ or Irish origin, to wean a greater number of lambs over the period of the study in comparison to Low Irish ewes. It may also be contributing to the fact that NZ ewes produced lambs of a heavier weaning weight than the Low Irish ewes, potentially indicating the utilization of body reserves to produce milk, during the lambing to week 6 PL phase, where lambs are reliant on ewe milk for energy (Fetherstone et al., 2021b).

An increased focus needs to be placed on breeding lighter ewes capable of producing heavier progeny in order to increase farm efficiency via lamb output per ewe and per hectare (Keady et al., 2009; McHugh et al., 2018). Previous beef and dairy studies have demonstrated that cows selected on the €uro-star Replacement index and Economic Breeding Index (high genetic merit) were lighter than those of low genetic merit (McCabe et al., 2017; O′Sullivan et al., 2020), while cows of NZ origin were also lighter than their Irish counterparts (Horan et al., 2005). Although differences were anticipated due to the large relative negative emphasis placed on ewe mature weight in Ireland (15.9%; Bohan et al., 2019) and in NZ (19.3%; Santos et al., 2015), results reported herein show no differences in ewe BW at any time point, between the three experimental groups, regardless of genetic merit or country of origin. Potentially greater differences could have been detected if more ewes were included within the study or the study was repeated over a greater number of years. It is noteworthy to mention that while there was no difference in ewe mature weight across the genetic merit groups, differences were reported for the trait that examined the proportion of litter BW produced at weaning relative to ewe BW at mating, where NZ ewes produced a greater proportion than Low Irish ewes. As a greater quantity of lamb was weaned without increasing the BW of the ewe herself, this may be an indication of potential to increase the efficiency of the national flock through the widespread use of genetic indexes in the future. This is a useful efficiency parameter widely reported in the past where results are consistent with those in this study (Earle et al., 2017a; McHugh et al., 2018). Findings from this study demonstrate the superior ability of NZ ewes to produce a greater proportion of litter BW at weaning relative to their own BW at mating, when compared with the Low Irish ewes. This further supports previous research that indicates the ability of lambs from high genetic merit ewes to grow faster and to reach slaughter targets at a younger age (Fetherstone et al., 2021b).

Furthermore, over the course of the study, fluctuations in the genetic merit of animals occurred, where 26% and 52% of ewes remained ranked within the top 20% (High genetic merit) and bottom 20% (Low genetic merit) for maternal genetic merit, respectively, by the end of the study. This indicates the change brought about as a result of the introduction of across-breed genomic evaluations (Pabiou et al., 2019), which impacted the ranking of animals when compared with others of another breed. Mature weight of lactating females is often used as a proxy for feed intake (Veerkamp and Thompson, 1999) and included in genetic indexes across beef, sheep, and dairy, as feed intake is a more difficult trait to measure on a large cohort of animals. Consequently, within genetic indexes, an assumption is often made that animals of the same BW have similar DMI; however, findings from this study contradict this during late lactation and highlight the need for more accurate DMI data to be incorporated into sheep indexes in the future.

Ewe efficiency can be further demonstrated by reporting milk yield and milk solids relative to ewe BW, in this case at week 6 PL. Such milk performance data have rarely been collected from nondairy sheep production systems (Ünal et al., 2008) or available with corresponding records, such as DMI, BCS, and ewe BW, as reported in this study. Milk yield at week 6 PL was greater for the NZ ewes in comparison to Irish ewes, and although no statistical differences were observed for ewe BW throughout the production year, when milk production was reported on per kilogram of ewe BW at week 6 PL basis, results indicated small biological differences in ewe BW as there was no difference in the ability of the three genetic merit groups to produce milk volume. However, NZ ewes produced a greater quantity of milk solids in comparison to the High Irish ewes per kilogram ewe BW at week 6 PL driven by their higher milk fat percentage. The difference in the fat content of milk between NZ and Irish ewes at week 6 PL indicated possible differences in fiber digestibility, volatile fatty acid production, de novo synthesis of milk fat, or mammary gland development that warrant further investigation, given the lack of mobilization of body reserves but the ability to produce milk of a higher fat content. Overall, ewe milk yield was similar to that previously predicted in Ireland by McGovern et al. (2015) and Campion et al. (2016), greater than previously reported internationally by Van der Linden et al. (2010), and surprisingly had a greater yield than recorded abroad for dairy ewes by Gonzalo et al. (1994) and Papadopoulos et al. (2002), albeit measured using alternative techniques. As previously highlighted by Snowder and Van Vleck (2003), ewe milk yield is known to be the main driver of lamb growth in the preweaning period. Differences in milk quantity and composition reported within this paper, whereby NZ ewes were superior to both High and Low Irish ewes, are in line with the lamb growth findings presented previously by Fetherstone et al. (2021b) that showed NZ lambs grew faster than both Irish groups of lambs from birth to drafting for slaughter. Albeit there were no differences detected across all genetic merit groups, both the percentage of milk protein and lactose reported in this study were in line with previous studies (Leitner et al., 2003; Afolayan et al., 2009). New Zealand ewes produced milk of a greater gross energy content (MJ/kg fresh milk) at week 6 PL in comparison to either group of Irish ewes, which is unsurprising given that milk fat content is the main driver of energy content in milk (Šebek and Everts, 1993). Toni et al. (2011) highlighted that a higher milk fat:protein ratio indicated a higher milk yield, which corroborates the findings of the present study where milk fat:protein ratios were 1.27:1, 1.12:1, and 0.96:1 for NZ, High Irish, and Low Irish ewes, respectively. Furthermore, Toni et al. (2011) demonstrated that cows with a milk fat percentage that was lower than their protein percentage would be predisposed to culling, disease, and lower milk production, similar to results reported for the Low Irish ewes (Fetherstone et al., 2021c).

A study by Kelly et al. (2020) demonstrates the ability to reduce DMI through the selection of high genetic merit beef animals on the terminal index, while maximizing carcass output; similar findings to this study where ewes of high genetic merit, whether of NZ or Irish origin, had a lower DMI in late lactation yet their lambs reached slaughter targets at a younger age (Fetherstone et al., 2021b). Furthermore, findings from this study suggest that careful selection of high genetic merit ewes within a flock could potentially allow increased stocking rates on large scale farms in late lactation; where low genetic merit ewes had 11% and 13% higher DMI per kilogram of ewe BW than NZ and High Irish ewes, respectively. This is in agreement with McCabe et al. (2017) who showed that low genetic merit beef cows have a 17% greater DMI per kilogram BW than high genetic merit beef cows. Even though no intake trait is included within the €uro-star replacement index, results from this study demonstrate that selecting high genetic merit ewes could possibly be indirectly selecting for lower DMI and improved DMI per kg of ewe BW, most likely due to the strong correlations reported between DMI and other traits such as milk yield (*r* = 0.78) and fertility (*r* = 0.50) in dairy cows (Bilal et al., 2016).

The greater BCS observed at mating, mid-pregnancy, lambing, and week 6 PL within this study may be a contributing factor to the increased reproductive performance (Fetherstone et al., 2021c), greater milk yield, milk solid content (Table 2), subsequent lamb performance (Fetherstone et al., 2021b), and overall efficiency (Figure 3) of the NZ ewes. High Irish ewes demonstrated a similar level of production efficiency to that of the NZ ewes for a number of traits including DMI parameters and their ability to wean a proportion of lamb BW closer to their own BW at mating, but the performance of High Irish ewes did not always differ to that of the Low Irish ewes. Although no difference in ewe BW was observed between genetic merit groups at any time point throughout the production year, the superiority of NZ ewes for the efficiency traits describing the proportion of litter BW weaned as a proportion of the ewe’s own BW at mating, and the quantity of milk solids produced per kilogram ewe BW at week 6 PL was as expected, that is, greater for NZ ewes than the Low Irish ewes. Compared with NZ ewes, the Low Irish ewes had a similar DMI at week 5 PL, while producing less milk at week 6 PL, and also gained BCS between lambing and weaning, highlighting their inefficiency whereby they utilize their energy intake during lactation to gain body reserves rather than convert it into litter BW via milk production. Further research into the comparison of economic breeding values (EBVs) and on-farm efficiency parameters should be carried out in the future, for example, does the proportion of litter BW weaned as a proportion of ewe BW increase through the selection of ewe mature weight, daughter milk, or days to slaughter. Furthermore, previous research carried out by Santos et al. (2015) demonstrated the similarity of the indexes in NZ and Ireland, particularly the maternal indexes, which were strongly correlated (0.86). It is possible that through outdoor lambing systems and more extensive production that NZ producers may have inadvertently selected toward more productive or efficient animals, that is, less assistance offered to ewes at lambing in outdoor systems in NZ could have lead to the selection of a more resilient, vigourous, easy-care replacements over time. 

## Conclusions

Ewes of New Zealand origin demonstrated their suitability in an Irish production system and ability to impact on-farm performance. High Irish ewes achieved similar results to NZ ewes, while differences between High and Low Irish ewes were not apparent within a number of traits, indicating the benefit of the use of high genetic merit animals but the need for continuous development of the genetic indexes in the future. Overall, results from this study could potentially lead to increased production and efficiency on sheep farms, as a result of an increase in the proportion of animals of high genetic merit being selected for breeding, now that the benefit of their use has been realized within this study. Therefore, potential to increase output, achieve superior conversion efficiency and/or energy utilization, and thereby improve flock productivity and profitability exists.

## Data Availability

The data and models reported within this study are available from the corresponding author upon reasonable request.

## Abbreviations

AI: artificial insemination
BCS: body condition score
BW: body weight
DMI: dry matter intake
MS: milk solids
PL: post-lambing
